# Spatial inequalities and predictors of HIV/AIDS mortality risk in Hamadan, Iran: a retrospective cohort study

**DOI:** 10.4178/epih.e2018038

**Published:** 2018-08-05

**Authors:** Somayeh Momenyan, Amir Kavousi, Jalal Poorolajal, Narges Momenyan

**Affiliations:** 1 Department of Biostatistics, Paramedical Sciences Faculty, Shahid Beheshti University of Medical Sciences, Tehran, Iran; 2 Department of Epidemiology, School of Public Health and Safety, Shahid Beheshti University of Medical Sciences, Tehran, Iran; 3 Research Center for Health Sciences and Department of Epidemiology, School of Public Health, Hamadan University of Medical Sciences, Hamadan, Iran; 4 Department of Medical Informatics, Tarbiat Modares University, Tehran, Iran

**Keywords:** HIV/AIDS, Mortality, Survival analysis, Spatial correlation, Iran

## Abstract

**OBJECTIVES:**

Understanding the geographic variation of HIV/AIDS mortality risk and human immunodeficiency virus (HIV) infection could help identify high-burden areas. The aim of our study was to evaluate the effects of predictors of the time interval between HIV diagnosis to death, while accounting for spatial correlations across counties, and to assess patterns of spatial inequalities in the risk of HIV/AIDS mortality in Hamadan Province, Iran.

**METHODS:**

This retrospective study was conducted on 585 patients. The outcome in this study was the time period between the date of HIV/AIDS diagnosis and the date of death. A Weibull regression model with spatial random effects was used.

**RESULTS:**

According to multivariate analysis, there were significant associations between age, tuberculosis co-infection, and marital status and the risk of death. In terms of spatial inequalities, a cluster of counties was identified with a somewhat higher death hazard in the north, northwest, northeast, and central regions. Additionally, a cluster with a somewhat lower hazard was identified in the south, southwest, southeast, and west regions.

**CONCLUSIONS:**

The spatial pattern of HIV/AIDS death risk could reflect inequalities in access to antiretroviral therapy and public health services. Our results underscore the importance of attention to vulnerable groups in urban areas.

## INTRODUCTION

Human immunodeficiency virus (HIV) infection has remained a public health problem despite extensive efforts to mitigate its impacts. Throughout the world, 75 million people have been infected with HIV and 35 million have died of acquired immunodeficiency syndrome (AIDS), as reported by the World Health Organization [1]. Fortunately, the annual number of AIDS-related deaths worldwide has gradually reduced from 2.3 million in 2005 to 1.5 million in 2013, with the introduction of antiretroviral therapy (ART) [2,3].

According to the place vulnerability theory, adverse life conditions do not affect all areas equally, and some areas are more vulnerable to disease and death than others [4]. Various studies have revealed that health outcomes, mortality, and disease outcomes depend on socioeconomic conditions and geographic areas [5,6]. For example, it has been shown that poor urban areas are more likely to be vulnerable to disease because of a lack of public health services and inadequate medical care. HIV/AIDS patients have a weakened immune system, which makes them vulnerable to opportunistic diseases, such as tuberculosis (TB), meningitis, bacterial pneumonia, encephalitis, and specific cancers [7]. Previous research has shown that HIV/AIDS mortality is related to socioeconomic status and residency [8]. Furthermore, numerous studies have shown that health insurance, which is related to socioeconomic status, plays an important role in the survival of HIV patients. Patients who are insured survive longer than those who lack health insurance, because they are more likely to have access to medical services [9-11]. Understanding the geographic variation of HIV/AIDS mortality and HIV infection could help identify high-burden areas. Thus, instead of distributing resources homogeneously within a country, resource allocation could be optimized.

To our knowledge, no study has investigated the spatial inequality in the risk of HIV/AIDS mortality in Iran. Because of that, the aim of our study was to examine the effect of predictors on the time interval between HIV diagnosis and death, while accounting for spatial correlations across counties, and to evaluate spatial inequality in the risk of HIV/AIDS mortality in Hamadan Province, which is located in the central-western part of Iran.

## MATERIALS AND METHODS

### Data description

This retrospective cohort study was conducted in Hamadan Province, Iran, from 1997 to 2011. The population of this study was people with HIV/AIDS who had a medical record in HIV testing and treatment centers of Hamadan Province. In these clinics, all services are performed anonymously, and patients do not need to provide their names. All 585 patients with HIV/AIDS who registered in these clinics were included in this study. The data collection consisted of demographic information (age at the first visit, sex, and marital status), method of transmission (injection drug use [IDU], sexual [heterosexual + male who have sex with male], mother to child, IDU/sexual, unknown), co-infection with TB, date of HIV/AIDS diagnosis, and date of death (if any). Information about vital status was checked through December 31, 2011, using active contact with patients or their family members.

A case of HIV in the Islamic Republic of Iran is defined as an individual for whom 2 sequential enzyme-linked immunosorbent assay tests are positive for the HIV antibody, followed and confirmed by a western blot test. A case of AIDS was defined as a definitive diagnosis of stages 3 or 4 conditions and/or a CD4 count less than 350/mm^3^ of blood in an HIV-infected subject [12]. AIDS diagnosis at baseline was defined by the incidence of an AIDS-defining disease at or before cohort registration.

The outcome in this study was the time period between the date of HIV/AIDS diagnosis and the date of death (available if death happened before December 31, 2011). Patients who did not die or were lost to follow-up were considered as censored. Accordingly, in the final outcome classification, patient cases were categorized into 2 categories: those who died and those who were censored.

### Statistical analysis

The outcome of interest in this study was the time interval between the time of HIV/AIDS diagnosis and death (in years). The 2effects of predictors were contrasted between the 2 groups (alive or lost to follow-up and AIDS or non-AIDS-related death) by the chi-square test. For the determination of the effects of predictors on the time to death, a Weibull regression model was used. Estimated cumulative hazards log (H) were plotted against the natural logarithm of time, and the presence of parallel lines suggested that the Weibull distribution would be appropriate for modeling the survival distribution. We considered spatial random effects in the regression model because the patients came from different counties, and patients within the same county had common health services or environmental risk factors. However, introducing spatial random effects accounts for possible differences in risk of death among the counties. Such spatial models are usually grouped into 2 general categories according to the data structure: point-referenced (geostatistical) data, where the exact geographic locations (e.g., latitude and longitude) are used, and areal (lattice) data, where the region of study is divided into a number of areal units with well-defined boundaries and the positions of each unit relative to each other are used [13]. In this study, we used the lattice approach, such that only information about the adjacency of each county to other counties was used. Finally, we mapped the spatial risk of mortality in each county. In our model, we assumed that *n* sample patients were from *K* counties, and *W_k_*, that indexed by *k*=1, …, *K*, denoted the spatial random effect for the *k*-th region. The intrinsic univariate conditionally autoregressive distribution was assigned to the spatial effects. More precisely, we formed *W~ N(0,[τ(D-B*)]^-1^), where *D*=Diag(m_k_), m_k_ is the number of neighbors of the *k*-th county, and *B* is the adjacency matrix of the graph representing our region (B_ii_=0,B_ij_=1 if region *i* is a neighbor of region *j*). Thus, the Weibull hazard function with the spatial random effects was as follows:

hiktXik=ρtρ-1expXikTβ+Wk

The variable *ρ* refers to the shape parameter. The Bayesian method was used to estimate all the model parameters. The multivariate normal and gamma priors were considered for regression coefficients and the Weibull shape parameter, respectively. For each predictor factor, the mean, standard errors, and unadjusted and adjusted hazard ratios (HRs) with 95% credible intervals (CIs) were obtained by the posterior samples. All statistical analyses and mapping of the results were performed using OpenBUGS and GeoBUGS version 3.2.3 (http://www.openbugs.net/w/FrontPage).

## RESULTS

A total of 585 registered patients with HIV/AIDS were included in the analysis, of whom 521 (89.1%) were males and 64 (10.9%) were females. Twenty-nine patients had been diagnosed with AIDS at their first visit to the clinic. The mean (standard deviation [SD]) age at the first visit was 32.59 (8.71) years. The majority of patients were aged 25 to 44 years and acquired HIV through injections (Table 1). In the final outcome classification, 403 patients (68.9%) were alive or lost to follow up, while 182 (31.1%) patients had died, either from AIDS or non-AIDS-related causes.

Our results showed significant differences in the distribution of mortality between sexes (p<0.001), across the 3 age groups that we analyzed (p=0.012), and according to co-infection with TB (p<0.001) and the mode of transmission (p=0.001). Specifically, the probability of death was higher among malemen, patients aged 45 to 74 years, and those who acquired HIV through injections (Table 1). However, the proportion of death did not show statistically significant variation according to marital status (p=0.13).

The effects of predictors on the HR of death, while accounting for spatial correlations across the counties, are shown in Table 2. From the posterior estimates of unadjusted HRs based on the spatial Weibull regression model, there were significant relationships between age at diagnosis, TB co-infection, and marital status and risk of death. In addition, based on the adjusted HR estimates, there were significant relationships between age at diagnosis, TB co-infection, and marital status with the risk of death. However, the adjusted relationship for risk of death was not statistically significant for the sex and mode of transmission. In other words, based on multivariate analysis, the risk of death was higher in patients aged 45 to 74 years than in those aged 0 to 24 years, such that the adjusted HR was 2.04. Moreover, HIV-positive patients who were co-infected with TB had a higher risk of death than those who were infected with HIV alone.

**Figure 1.**
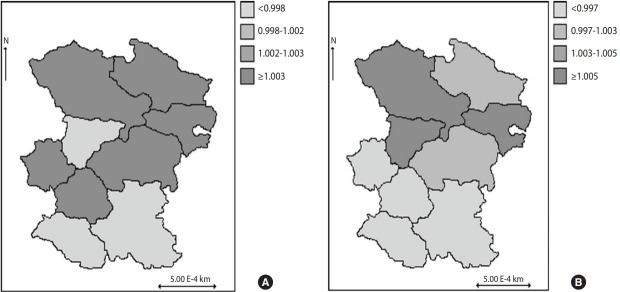
Maps of the HIV/AIDS death risk in a cohort study in Hamadan, Iran (1997-2011) by a spatial Weibull regression model without covariates (A) and with covariates (B).

We also mapped summaries of our results. Figure 1 shows 2 maps that represent the posterior spatial HR defined by exp(*W_k_*) in 9 counties of Hamadan Province based on a spatial Weibull model. Figure 1A was generated using a model that did not consider the effects of covariates, and considered only the unobserved spatial variation, whereas Figure 1B was developed using a model that considered both the effects of covariates and spatial random effects. To show the spatial disparities on the map, the posterior estimates of spatial random effects for each county were recorded based on the quartile of their distribution. Based on the map Figure 1A, the counties with a lower risk of death were located in south, southeast, southwest, and west of the Hamadan Province (3 of the 9 counties). Contrastingly, the north, northeast, northwest, and central regions were higher-risk regions (6 of the 9 counties). The map Figure 1B repeats this mapping for the model, but includes all covariates. It should be noted that when considering the effects of covariates along with spatial random effects, the visualized results were similar to those on the Figure 1A. As shown in Figure 1, for the risk of death, a cluster of counties was identified with somewhat higher hazards in the north, northwest, northeast, and central regions (5 of the 9 counties), as well as a cluster with somewhat lower hazards in the south, southwest, southeast, and west regions (4 of the 9 counties). The values of spatial random effects for risk of death ranged from -0.005 to 0.005, based on the model with covariates. Such small values of the spatial random effects suggest that regional differences had a small effect on the risk of death, although the values of spatial random effects could help explain risk inequality.

## DISCUSSION

The majority of patients became infected with HIV through drug injection. Thus, it is essential to focus attention on this group in order to reduce HIV transmission risk and the time between HIV infection and diagnosis. In our study based on a spatial Weibull regression model, there were significant associations between age at diagnosis, TB co-infection, and marital status and the risk of death. In other words, the risk of death was higher in patients of 45 to 74 years and patients who were co-infected with TB. These findings agree with those of recent reports of HIV cohort studies. Previous studies have shown that patients aged 50 years or above were at a higher risk of death than younger patients [14-16]. Poorolajal et al. [17] reported that the risk of AIDS-related mortality was twice as high in patients with TB than in those infected with HIV alone. Our study found a cluster with a higher risk of death in the north, northwest, northeast, and central regions of Hamadan Province, as well as a cluster with a lower risk of death in the south, southwest, southeast, and west regions. The cluster with a higher death risk contained counties with lower population density than other counties. The degree of development of healthcare resources was reported to be lower in the counties of this cluster than in the other counties using the numerical taxonomy technique. However, the results of our study can be explained based on the possibility that individuals who live in remote areas with a lower population density are less likely to use free HIV care provided by government programs than others. However, the lower-risk cluster contained some counties with a higher population density. This is unlike earlier research, which reported lower survival in remote areas and higher survival in urban areas [18]. However, the results of our study can be explained based on the possibility that individuals who live in remote areas with a lower population density are more likely to use free HIV care provided by government programs than others. Earlier studies have evaluated spatial disparities in HIV/AIDS data. Oppong & Harold [4] assessed survival of HIV/AIDS cases by socioeconomic status between 1999 and 2008 in Dallas, TX, US. They showed that areas with high socioeconomic status had higher survival rates than those with low socioeconomic status [4]. Jongstepongpanth & Bagchi-Sen [19] reported spatial variations in the risk of HIV/AIDS death between 2000 and 2004 in Thailand. Martins et al. [18] assessed the risk of HIV/AIDS death in Brazilian states from 2002 to 2006. They showed that the Brazilian states with a higher risk of death were located in the north and northeast regions, and were more distant from the most populous states in Brazil, which is contrary to our results. However, they found small variance for spatial random effects at the state level in Brazil, which is in concordance with our results. It is possible that the variance in spatial random effects is not statistically significant, but that in spatial survival analysis, the length of the 95% CIs for covariates is reduced. In other words, spatial models may yield better predictions than independent models [18]. Our study had strengths and limitations that should be considered. The first strength is that the vital status of all patients was checked through December 31, 2011. The first limitation of our study is its retrospective design. The second limitation is that some covariates, such as ART status and viral load, were not included in the analysis. Third, 29 patients were diagnosed with AIDS at the first visit. This may have led to an underestimation of the actual survival probability from HIV infection to death.

In conclusion, the spatial pattern of HIV/AIDS mortality risk could reflect inequalities in access to ART and public health services. Our results underscore the importance of considering vulnerable groups in urban areas by offering free government programs.

## Figures and Tables

**Table 1.** Predictor factors of the patients by their final outcome in a cohort study in Hamadan, Iran (1997-2011)

Variable	Alive or lost to follow-up (n=403)	AIDS or non-AIDS-related death (n=182)	Total (n)	p-value
Sex				<0.001
Male	344 (66.0)	177 (34.0)	521	
Female	59 (92.2)	5 (7.8)	64	
Marital status				0.13
Single	194 (72.4)	74 (27.6)	268	
Married	149 (66.8)	74 (33.2)	223	
Divorced	45 (60.0)	30 (40.0)	75	
Widow	15 (78.9)	4 (21.1)	19	
Age (yr)				0.01
1-24	54 (76.1)	17 (23.9)	71	
25-44	319 (69.8)	138 (30.2)	457	
45-74	30 (52.6)	27 (47.4)	57	
Tuberculosis infection				<0.001
No	396 (70.2)	168 (29.8)	564	
Yes	7 (33.3)	14 (66.7)	21	
Mode of HIV transmission^1^				0.001
Injection drug use	310 (65.3)	165 (34.7)	475	
Sexual	62 (86.1)	10 (13.9)	72	
Mother to child	8 (88.9)	1 (11.1)	9	
Injection drug use/sexual	21 (80.8)	5 (19.2)	26	

Values are presented as number (%). AIDS, acquired immunodeficiency syndrome; HIV, human immunodeficiency virus.

1 There are missing values for 3 individuals.

**Table 2.** Results of the spatial Weibull regression model for the risk of HIV/AIDS death in a cohort study in Hamadan, Iran (1997-2011)

Variable	Unadjusted estimate		Adjusted estimate	
	Mean±SD	HR (95% CI)	Mean±SD	HR (95% CI)
Sex				
Male		1.00 (reference)		1.00 (reference)
Female	-1.56±0.48	0.23 (0.07, 0.48)	-1.41±0.58	0.28 (0.07, 0.70)
Marital status				
Single		1.00 (reference)		1.00 (reference)
Married	0.30±0.16	1.37 (0.97, 1.86)	0.35±0.17	1.44 (1.01, 2.00)^*^
Divorced	0.57±0.21	1.81 (1.14, 2.68)^*^	0.44±0.23	1.59 (0.97, 2.41)
Widow	-0.13±0.54	0.99 (0.26, 2.23)	0.05±0.55	1.21 (0.31, 2.77)
Age (yr)				
1-24		1.00 (reference)		1.00 (reference)
25-44	0.36±0.26	1.49 (0.89, 2.47)	0.07±0.26	1.12 (0.65, 1.88)
45-74	1.08±0.31	3.12 (1.62, 5.61)^*^	0.67±0.32	2.07 (1.04, 3.81)^*^
Tuberculosis infection				
No		1.00 (reference)		1.00 (reference)
Yes	0.71±0.28	2.13 (1.13, 3.46)^*^	0.67±0.29	2.04 (1.07, 3.37)^*^
Mode of transmission				
Injection drug use		1.00 (reference)		1.00 (reference)
Sexual	-0.86±0.33	0.44 (0.20, 0.78)	-0.29±0.39	0.80 (0.32, 1.53)
Mother to child	-1.75±1.23	0.30 (0.01, 1.16)	-0.30±1.34	1.42 (0.03, 5.95)
Injection drug user/sexual	-0.52±0.47	0.65 (0.21, 1.35)	-0.25±0.48	0.86 (0.27, 1.81)

SD, standard deviation; HR, hazard ratio; CI, credible interval.

* p<0.05.
